# Population Pharmacokinetics of Lithium in Young Pediatric Patients With Intellectual Disability

**DOI:** 10.3389/fphar.2021.650298

**Published:** 2021-04-15

**Authors:** Junying Yuan, Bohao Zhang, Yiran Xu, Xiaoli Zhang, Juan Song, Wenhao Zhou, Kai Hu, Dengna Zhu, Lirong Zhang, Fengmin Shao, Shusheng Zhang, Junjie Ding, Changlian Zhu

**Affiliations:** ^1^ Henan Key Laboratory of Child Brain Injury and Henan Pediatric Clinical Research Center, Institute of Neuroscience and Third Affiliated Hospital and of Zhengzhou University, Zhengzhou, China; ^2^ College of Chemistry and Molecular Engineering, Zhengzhou University, Zhengzhou, China; ^3^ Department of Pediatrics, Children’s Hospital of Fudan University, Shanghai, China; ^4^ Academy of Chinese Medical Sciences, Henan University of Chinese Medicine, Zhengzhou, China; ^5^ Department of Pharmacology, School of Basic Medical Sciences, Zhengzhou University, Zhengzhou, China; ^6^ Henan Key Laboratory of Kidney Disease and Immunology, People’s Hospital of Zhengzhou University, Zhengzhou, China; ^7^ Centre for Tropical Medicine and Global Health, Nuffield Department of Clinical Medicine, University of Oxford, Oxford, United Kingdom; ^8^ Department of Women's and Children's Health, Karolinska Institutet, Stockholm, Sweden; ^9^ Center for Brain Repair and Rehabilitation, Institute of Neuroscience and Physiology, University of Gothenburg, Göteborg, Sweden

**Keywords:** lithium, clinical pharmacokinetics, population pharmacokinetics, child, intellectual disability

## Abstract

**Background:** Lithium is a well-established treatment for bipolar disorders and has been shown to be neuroprotective, and thus low doses might be useful for the treatment of childhood brain injury and neurological sequelae. However, pharmacokinetic (PK) data in children are limited. This study was to investigate the PKs after oral administration of low-dose lithium carbonate in young children with intellectual disability.

**Methods:** Fifty-two children with intellectual disability aged 4–10 years old were enrolled. A series of blood samples were collected after a single-dose administration of lithium carbonate. The serum lithium concentration was measured using a validated ion chromatography assay, and the PK concentration data were modeled using a nonlinear mixed effect model in the NONMEM program.

**Results:** The lithium concentration over time was adequately described by a two-compartment disposition, with a transient absorption and first-order elimination process. The inclusion of body weight as an allometric factor significantly improved the model fit, but age and gender were not associated with the PKs of lithium. The clearance, central volume, inter-compartmental clearance, and peripheral volume estimates from the final population PK model were 0.98 L/h, 13.1 L, 0.84 L/h, and 8.2 L for children with a body weight of 20 kg. The model evaluation suggested that there is no obvious discrepancy between the observations and predictions in the proposed model. A visual predictive check demonstrated the good predictive performance of the final model.

**Conclusions:** The lithium PK properties in young children were similar to those in older children and adults. The proposed model can be used for further PK/PD analysis to optimize the dosage regimen of lithium in children.

## Introduction

Lithium is a widely used and effective treatment for individuals with psycho-neurological disorders, and it is still the benchmark treatment for bipolar disorders (Berk et al., 2017; Won and Kim, 2017; Baldessarini et al., 2019) with evidence of therapeutic benefit dating back almost 70 years (Cade, 1949). Recent studies have shown that lithium has multiple functions (Morlet et al., 2018), and it exhibits protective and regenerative properties in multiple animal brain injury models (Li et al., 2011; Huo et al., 2012; Contestabile et al., 2013; Xie et al., 2014). It has therefore been suggested that low doses of lithium might be useful for treating other neurological disorders such as stroke and neurodegenerative disorders (Mohammadianinejad et al., 2014; Matsunaga et al., 2015). Some studies have verified that lithium is beneficial for treating intellectual disability (Serret et al., 2015; Figueiredo et al., 2016; Yuan et al., 2018) and might be a treatment option for children with behavior problems (Malone et al., 2000; Amerio et al., 2018; Pisano et al., 2019), but the clinical experience in young children is limited due to potential toxicity (Shine et al., 2015). With the lack of pharmacokinetic (PK) guidelines for its use in pediatrics, lithium has been underused in pediatric populations (Grant and Salpekar, 2018).

Lithium salt forms (i.e., lithium carbonate) have a complete oral bioavailability (approximately 100%). Lithium does not bind with protein and distributes unevenly into different body compartments, and it is not metabolized and is eliminated primarily through the kidneys. The systemic lithium PK profile follows a two-compartment disposition model. The reported clearance is between 0.51 and 1.59 L/h, and the central distribution volume ranges from 15.2 to 39.7 L, with these parameters allometrically scaled to 70 kg body weight when possible (ElDesoky et al., 2008; Findling et al., 2010; Landersdorfer et al., 2017; Methaneethorn, 2018; Alqahtani et al., 2019).

Even though lithium has been used for several decades, the optimal dosage regimen is still a matter of some debate. Moreover, the current lithium dose regimen is mainly for bipolar disorder therapy, which generally needs high serum concentrations and therefore has a narrow therapeutic window of 0.6–1.2 mmol/L and a maintenance therapy window of 0.6–0.8 mmol/L (Methaneethorn and Sringam, 2019). The PK properties of lithium treatment in adults have been reported previously (Wing et al., 1997; Canal et al., 2003; Wong et al., 2011), but studies on the PKs of lithium in young pediatric patients are rare. One PK study in nine children aged 10–12 years and diagnosed with behavioral disorder or adjustment disorder receiving a single dose of 300 mg lithium carbonate suggested that the PK parameters were similar to those of adults, except for the higher total clearance in children (Vitiello et al., 1988). Another study reported PK data in 39 children with bipolar disorder (20 males and 19 females) aged 7–17 years old. The subjects all had a body weight of 20 kg or more and were randomly assigned to receive either a single 600 or 900 mg dose of lithium (Findling et al., 2010). All of these lithium PK studies were performed using a fixed dose of lithium, and the subjects were all aged 7 years or older (Vitiello et al., 1988; Wing et al., 1997; Findling et al., 2010). Furthermore, the body weight and age were reported to be correlated with the lithium clearance (Yukawa et al., 1993; Findling et al., 2010; Rej et al., 2014; Landersdorfer et al., 2017; Methaneethorn and Sringam, 2019). The extrapolation of PK profiles from bipolar disorders to developmental neurological disorders such as intellectual disabilities has high uncertainty, and the optimal therapeutic level of lithium is not well established. Taken together, the previous PK studies of lithium in pediatric populations have limitations (e.g., small sample size, not including young children, bipolar disorder indication). Again, the age-related maturation effect might affect the PK disposition of lithium in young children, and this might result in different PK profiles. Hence, the PKs in young children still need further evaluation.

The purpose of this study was to characterize the PK properties of lithium in younger pediatric patients with intellectual disability and to identify the significant covariates that might affect PK profiles. The results presented here provide support for further pharmacokinetic/pharmacodynamic (PK/PD) evaluations in young children.

## Methods

### Study Subjects

Young children aged 4–10 years old with intellectual disability according to the Diagnostic and Statistical Manual of Mental Disorders, Fifth edition were deemed eligible for this study (Papazoglou et al., 2014). The inclusion criteria were 1) intelligence quotient <70 as evaluated by the Wechsler Intelligence Scale for Children, Fourth edition, China Revised or the Wechsler Preschool and Primary Scale of Intelligence, Fourth edition, China Revised; 2) deficits or impairments in adaptive function with two or more skill areas; 3) normal laboratory tests such as liver function, renal function, routine blood analysis, electrocardiogram, and thyroid function; and 4) the ability to swallow a tablet alone or under the supervision of a parent/guardian. The exclusion criteria were 1) inherited metabolic disorder; 2) the use of any medication in the previous month that might be adversely affected by lithium or might influence the efficacy or safety of lithium; and 3) a history of allergies or adverse reactions to lithium. This study was approved by the Ethics Committee of the Third Affiliated Hospital of Zhengzhou University (2015/AFZZ/15), and the parents/guardians of all study subjects provided written informed consent before participation.

### Medication Dosing

Prior to lithium carbonate (Li_2_CO_3,_ Cat# 181,104, Hunan Qianjin Xiangjiang Pharmaceutical Industry Co., Ltd. China) administration, subjects were required to fast for at least 4 h. Eligibility criteria were reviewed and confirmed prior to receiving the medication. The body weight was recorded before lithium administration, and the dose amount was adjusted to 12 mg/kg body weight. If the subject vomited after taking the lithium carbonate, the subject was postponed from the trial and then included again after one week.

A total of 16 children (eight boys and eight girls) were enrolled in the intensive PK cohort from whom a series of serum samples were collected at 0.5, 1, 1.5, 2, 4, 8, 12, 24, 36, and 48 h after lithium carbonate administration. The remaining 36 children (30 boys, six girls) were enrolled in the sparse PK cohort from whom at least three serum samples were collected from among the above time points. The serum samples were stored at −80°C until lithium concentration measurement.

### Sample Preparation and Lithium Assays

Before the analysis, the serum was thawed and treated with nitric acid, and then heated to 100°C for 2 h until dryness. After that, the residue was redissolved with ultrapure water. Methanesulfonic acid (400 μl, 20 mmol/L) was added to the supernatant of the serum sample (100 μl), and the mixture was vortexed for at least 1 min. The solution was transferred into a Millipore Amicon ultra-centrifugation filter (0.5 ml, 3 kDa) and centrifuged at 14,000 × *g* for 30 min at 4°C. The solution was then passed through a 0.22 μm nylon syringe filter and subjected to ion chromatography analysis using a ThermoFisher Scientific ICS-5000 + system (Dionex, Sunnyvale, CA, United States) (Zerbinati et al., 2000) equipped with a Dionex CERS-500 suppressor (4 mm), an AS-AP autosampler, a conductivity detector, a DS6 heated conductivity cell detector, a tandem double piston reciprocating pump, and a Chromeleon 7.0 chromatography data system. A Dionex ionpac CS12 A (4.0 mm × 50 mm) was used as the guard column, and an analytical Dionex ionpac CS12A RFIC column (4.0 mm × 250 mm) was used for analysis at a flow rate of 1.0 ml/min under isocratic conditions at 30°C. The optimized chromatographic condition was obtained with 20 mmol/L methanesulfonic acid as the mobile phase. The precision of the method was validated by analysis of intra-day and inter-day relative standard deviation values in spiked sample solution, and these were in the ranges of 0.34–2.7% and 0.79–3.4%, respectively. The method’s accuracy was calculated at three concentration levels, and the recoveries of Li^+^ were found to be in the range of 98.3–108.6%. Each sample was measured three times, and the average was used as the value of the sample. The linear range of this assay was 1.44–72.05 μmol/L, and the limit of detection was 0.43 μmol/L.

The population PK analysis was performed using nonlinear mixed-effects modeling in the NONMEM software (version 7.4, ICON Development Solutions, Ellicott City, MD, United States) compiled with gFortran (version 4.60). Perl-speaks-NONMEM (version 4.6.0) and R (version 3.2.0, http://www.r-project.org/) were used to evaluate the goodness of fit and to visualize the outputs. The first-order conditional estimation method with η-ε interaction was used throughout the model-building procedure. Discrimination between models during the model-building phase was based on the objective function value (OFV), which was calculated as proportional to twice the log-likelihood of the data. A reduction in OFV (∆OFV) of 10.83 was considered a significant improvement (*p* < 0.001) between two hierarchical models after inclusion of one extra parameter (one degree of freedom difference).

Because lithium is an endogenous element, the pre-dose lithium concentration was deducted from the post-dose concentration. The lithium concentrations were transformed into their natural logarithms, and first-order elimination of lithium was assumed to occur from the central compartment. All possible structural distribution compartments were investigated, i.e. one-, two-, and three-compartment disposition models. The absorption of lithium was assumed to be a first order process. The delay in the absorption was modeled using different candidate models (e.g., lag time and a flexible transit absorption model by adding 1–10 transit compartments (Savic et al., 2007). Inter-individual variability was modeled exponentially for all PK parameters according to

$$\theta_{i}=\;\theta \cdot \exp\left(\eta_{i,\theta }\right)$$ (1)

where $\theta_{i}$ is the individual parameter estimate for the *i*th individual, θ is the population estimate of the PK parameter, and $\eta_{i,\theta }$ is the inter-individual variability (IIV) of the PK parameter, which is assumed to be normally distributed with a mean of zero and a variance of ω^2^. Relative bioavailability (F) was fixed to unity in the population in order to allow investigation of the IIV of absorption. The residual unexplained variability on log-transformed concentrations was modeled with an additive error (Equation 2, equivalent to an exponential residual error on an arithmetic scale), proportional error (equation 3, equivalent to an additive residual error on an arithmetic scale), and their combination (Eq. 4).

Y= LOG(F)+ε (2)

Y= LOG(F)+ε/exp(LOG(F)) (3)

$$Y=\;LOG\left(F\right)+\varepsilon_{1}+\;\varepsilon_{2}/\exp\left(LOG\left(F\right)\right)$$ (4)

where Y represents the observation and F is the individual prediction. The residual variability was assumed to be normally distributed with a mean of zero and a variance of σ^2^.

### Covariate Modeling

Body weight was included in the model as the simultaneous incorporation of an allometric function on all clearance and distribution volume parameters as respectively, where BW_i_ is the individual body weight and BW_median_ is the median body weight of the study population (i.e. 20 kg).

$$\theta_{i}=\;\theta \cdot {\left(\frac{BW_{i}}{BW_{median}}\right)}^{0.75}\cdot \exp\left(\eta_{i,\theta }\right)$$ (5)

and

$$\theta_{i}=\;\theta \cdot \left(\frac{BW_{i}}{BW_{median}}\right)\cdot \exp\left(\eta_{i,\theta }\right)$$ (6)

The effect of age-related maturation on the clearance was then evaluated using a saturation-type function (equation 7), as proposed the literatures (Holford et al., 2013; Ding et al., 2015). Where $CL_{i}$ is the individually predicted clearance and $CL_{STD}$ is the typical clearance value of the population. $Age_{50}$ is the age associated with reaching 50% of the clearance maturity.

$$CL_{i}=CL_{STD}\cdot \frac{Age}{Age_{50}+Age}\cdot {\left(\frac{BW_{i}}{BW_{median}}\right)}^{0.75}\cdot \exp\left(\eta_{i,CL}\right)$$ (7)

Finally, gender was investigated as categorical variable (male = 0, female = 1) for all model parameters using a proportional equation (Eq. 8). where $\theta_{STD}$ is the typical parameter value. $\theta_{sex}$ is the gender effect on the PK parameter.

$$\;\theta =\;\theta_{STD}\cdot \left(1+\theta_{sex}\cdot Sex\right)$$ (8)

### Model Evaluation

Basic goodness-of-fit diagnostics were used to evaluate systematic errors and model misspecification, and the sampling-importance resampling (SIR) approach was used to calculate the parameter uncertainty in the final population PK model. The SIR approach has been proposed to improve the estimation of parameter uncertainty for non-linear mixed-effect models compared to currently available methods like the asymptotic variance-covariance matrix and the bootstrap method, and SIR is much less computationally intense compared to the bootstrap method (Dosne et al., 2016). The overall predictive performance of the final model was evaluated using simulation-based diagnostics (i.e. visual predictive checks, n = 2,000 simulations).

### *In Silico* Simulations

The lithium concentration-time profiles at steady state (assuming patients administered lithium carbonate with a loading dose of 12 mg/kg body weight and then given 6 mg/kg body weight twice daily for 10 days) of 2,000 individuals were simulated under a number of scenarios including significant clinical covariates (e.g., body weight) if possible based on final model. The key PK exposure parameters (i.e., AUCtau, Cmax, and Ctrough) were summarized.

## Results

### Demographics

A total of 52 subjects (38 males and 14 females) received a single dose of oral lithium carbonate, and the PK samples were collected as planned. The average age and body weight were 84.8 ± 21.7 (48–128) months and 23.0 ± 6.2 (16–44) kg, respectively.

### Population PK

**FIGURE 1:**
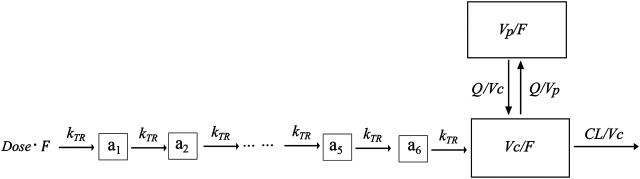
Graphical overview of the structural PK model for lithium. F is the relative bioavailability, CL is the elimination clearance, Vc is the central volume of distribution, MTT is the mean transit time, Vp is the peripheral volume of distribution, Q is the inter-compartmental clearance, and a1-a6 is the transit absorption compartment one to 6.

A total of 382 blood samples were available for lithium concentration measurements, and all of the plasma concentration measurements were above the LLOQ of 0.00144 mmol/L. The lithium concentration over time was fitted using a one-compartment model with a first-order absorption and elimination process (OFV = 84.067) with an additive residue error model. Implementation of a combined proportional and additive residue error model did not improve model fit significantly. The two-disposition compartment model improved the model fit (∆OFV = −24.01), and implementation of a transit absorption compartment (*n* = 6) improved the model fit even further (∆OFV = −261.553). The structure of the model is shown in Figure 1.

Body weight, implemented as a fixed allometric function for all clearance and volume of distribution parameters, showed a substantial improvement in model fit (∆OFV = −7.891). Including age-dependent maturation on clearance did not improve model fit further. Finally, gender did not have a significant impact on lithium PK properties.

The IIV estimate for the elimination clearance had poor precision with a relative standard error of 195%, and the IIV estimate for Q (the inter-compartmental clearance) was very close to zero. Therefore, these two IIVs were not included in the final model.

**FIGURE 2:**
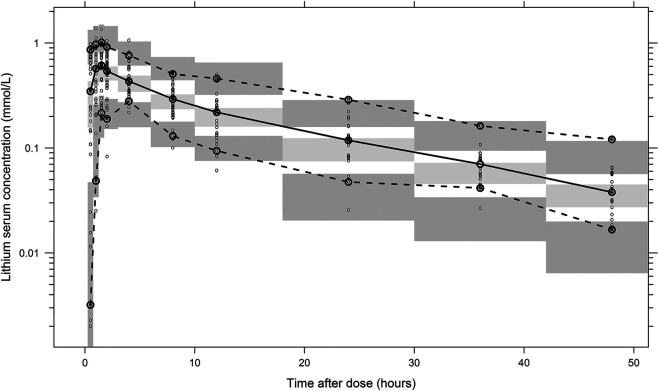
Visual predictive check of the final population PK model for lithium based on 2,000 stochastic simulations. Open circles represent the observed concentrations, and solid lines represent the fifth, 50th, and 95th percentiles of the observed data. The shaded areas represent the 95% confidence intervals around the simulated fifth, 50th, and 95th percentiles of the prediction.

**FIGURE 3:**
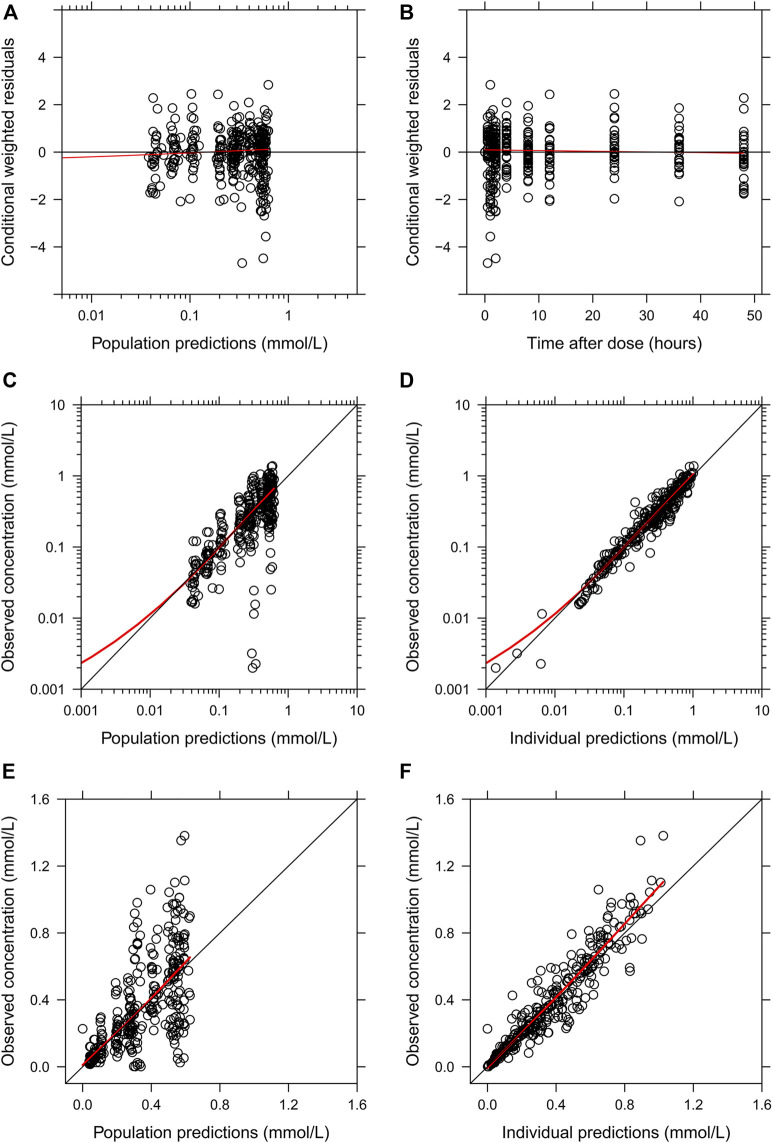
Goodness-of-fit plots of the final population PK model of lithium. **(A)** Conditional weighted residuals *vs.* population-predicted lithium concentrations. **(B)** Conditional weighted residuals *vs.* time. **(C,E)** Observed serum lithium concentrations *vs.* population-predicted concentrations in log and linear scales. **(D,F)** Observed serum lithium concentrations *vs.* individually predicted concentrations in log and linear scales. Solid red lines represent locally weighted least squares regressions.

The final parameter estimates showed good precision with relatively small standard errors (Table 1), thus confirming the stability of the model and providing confidence when using the developed population PK model to simulate different adherence scenarios. The final parameter estimates described the expected absorption, distribution, and elimination processes, as well as the associated unexplained variability of lithium in children. Visual predictive checks (Figure 2) and goodness-of-fit diagnostic plots (Figure 3) gave good descriptions of the observed data and demonstrated the adequate predictive performance of the final model.

**TABLE 1:** Final population PK parameter estimates of lithium in children.

Parameter	NONMEM estimates (%RSE)	SIR median (95%CI)	CV for IIV (%RSE)	SIR median (95%CI)	Shrinkage (%)
F (%)	100 *fixed*	—	30.3 (10.9)	30.5 (24.1–40.1)	11.6
MTT (h)	0.52 (9.9)	0.52 (0.40–0.71)	65.0 (13.1)	65.5 (50.6–85.4)	29.6
Number of transit compartment	6 *fixed*			—	—
CL/F (L/h)	0.98 (4.6)	0.98 (0.90–1.07)	—	—	—
V_C_/F (L)	13.1 (7.2)	13.2 (11.7–15.2)	26.5 (16.1)	27.2 (17.2–37.3)	27.1
Q/F (L/h)	0.84 (9.5)	0.84 (0.61–1.07)	—	—	—
V_p_/F (L)	8.2 (17.7)	8.3 (5.5–11.4)	118.7 (15.4)	118.2 (86.3–164.0)	35.4
σ	0.091 (19.0)	0.092 (0.076–1.11)	—	—	—

Note: F, relative bioavailability; MTT, mean transit time. CL/F,elimination clearance; V_C_/F, central volume of distribution; V_p_/F, peripheral volume of distribution; Q, inter-compartmental clearance; σ, additive residue error on a log scale. Population estimates in the table are given for a “typical” child with body weight of 20 kg and full maturation of metabolizing enzymes. Coefficients of variation for inter-individual variability (IIV) were calculated as 100 × (e^variance^)^1/2^. Relative standard errors (%RSE) were calculated as 100 × (standard deviation/mean). SIR: The sampling-importance resampling method. The uncertainty was derived from SIR with options of 2,000 samples and 1,000 resamples. NONMEM: nonlinear mixed effect model.

### Simulation Under Clinical Scenarios

**FIGURE 4:**
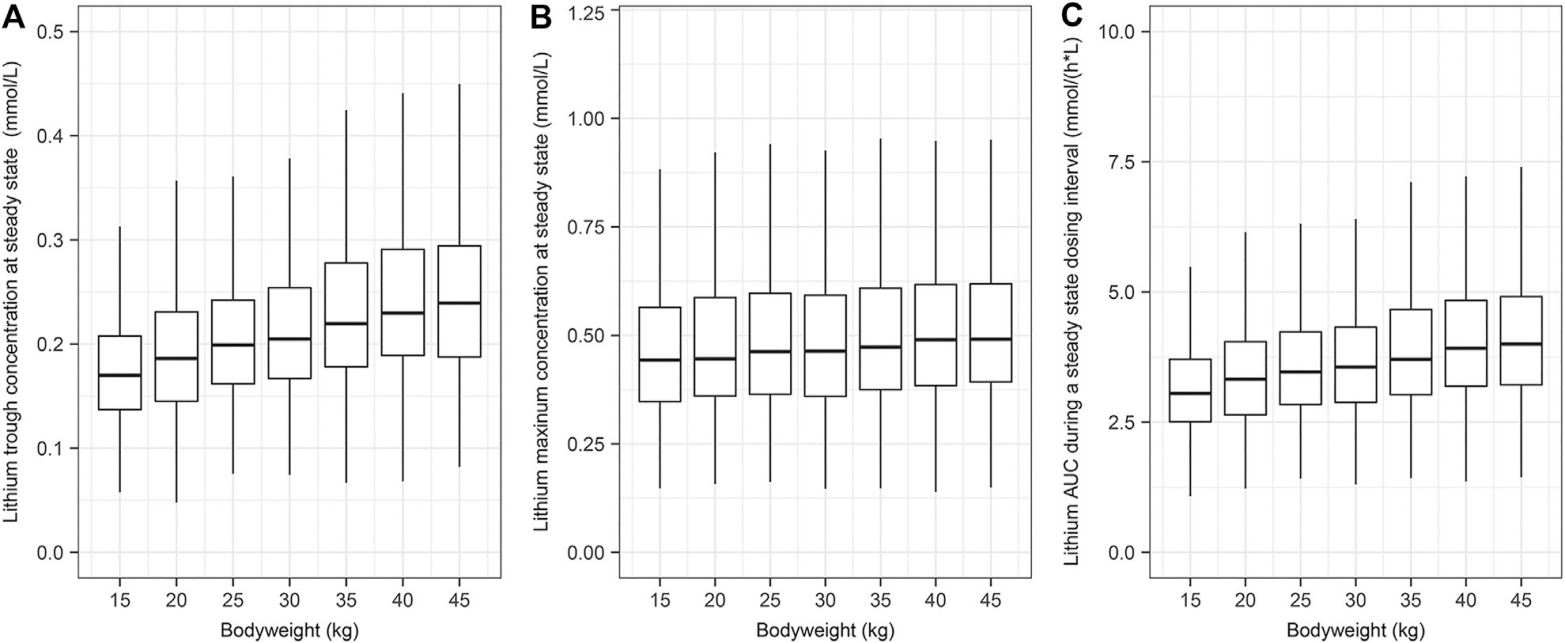
The simulated systemic lithium exposures at steady state. A total of 1,000 children were simulated for each body weight group. The children were assumed to receive a loading dose of 12 mg/kg of lithium carbonate and then 6 mg/kg every 12 h for 10 days. **(A)** Lithium trough concentration at steady state. **(B)** Lithium maximum concentration at steady state. **(C)** Lithium AUC during a steady state dosing interval.

Body weight was identified as a significant covariate and was considered in the simulation scenarios. The simulated children were administered lithium carbonate with a loading dose of 12 mg/kg and then given 6 mg/kg twice daily for 10 days to ensure that the PK reached the steady state. The concentration-time data of 1,000 individuals in each scenario were simulated based on the final population PK model. The drug exposures (i.e., trough concentration, C_max_, and AUC_0-t_) were decreased along with increased body weight, as demonstrated in Figure 4.

## Discussion

Lithium is one of the most frequently used mood stabilizers, but it has not been recommended for use in non-psychological disorders in children because of the potential toxicity of the dosage regimen used for bipolar disorder, even though multiple studies have shown beneficial effects (Zyoud et al., 2017; Pisano et al., 2019). The current study reports the PK properties of lithium in a large young pediatric population (n = 52), of whom 16 children had intensive PK samples collected. This study provides support for further PK/PD assessments in these patients.

Lithium carbonate is the most commonly used lithium salt, and the absorption is slower than for other salt forms (Grandjean and Aubry, 2009). The PK properties are critical for informing individualized therapeutic regimens in the target population, but no PK studies have been performed in children younger than 7 years old. It has been shown that lithium clearance is age related (Findling et al., 2010), and thus it is necessary to investigate the PK profiles in young children taking low-dose lithium for the treatment of neurodevelopmental disorders (Yuan et al., 2018).

In the current study, the clearance estimate was 0.98 L/h for a typical child with a body weight of 20 kg, which is slightly higher than previous reports of 0.67 L/h and 0.63 L/h for a 20 kg child with bipolar disorder (Findling et al., 2010; Landersdorfer et al., 2017). This bodyweight normalised clearance value (0.049 L/kg/h) was also higher than that reported for acute mania patients [0.022 L/kg/h (1.43 L/h for a 65 kg adult)] and for bipolar disorder patients [(0.014 L/kg/h (1.15 L/h for an 82 kg adult)]. The slightly higher clearance in the current study might be attributed to the PK variability between different populations and indications as well. This also might be associated with different PK sampling strategies across studies. In current study, some patients collected PK sample over a short period (up to 24 h), which could affect clearance estimate. Again, the average half-life estimate in the current analysis was 19.1 h, which was consistent with the reports of 11–20 h in previous studies (Shelley and Silverstone, 1986; Vitiello et al., 1988).

Achieving high lithium concentrations in the blood is the goal for treating bipolar disorders, but high lithium concentrations can have toxic effects and thus therapeutic monitoring of lithium concentrations is compulsory with high doses for bipolar disorders. Our simulation based on the clinical dosage regimen suggests that the lithium plasma exposure C_max_ was 0.47 mmol/L with a 95% confidence interval of 0.23–0.99 mmol/L, which was lower than the lower limit of toxicity (1.2 mmol/L) (Chehovich et al., 2020). This indicates that this lithium regimen for children with non-bipolar disorder was below the safety threshold with a median maximum concentration of 0.47 mmol/L. Importantly, this dosage regimen was found to be safe in the study population, and thus it might not be necessary to monitor the lithium concentration with such a low-dose treatment in children with normal kidney function (Yuan et al., 2018).

There are some limitations of this study that should be noted. First, the subjects were children with intellectual disability from a single center and with an age of 4–10 years, and thus did not cover all age groups of children. The PK data reported here should be applied with caution to other age groups of children or in generalizing to the normally developing children because the absorption, distribution, metabolism, and excretion of lithium can vary in other age groups. Second, this study was limited to inpatient children with intellectual disability, and the PK data can vary significantly compared with healthy children, even though the data reflect clinical applications and are more useful for developing personalized treatments. Third, there were more boys than girls in this study, which might have produced gender-related bias, although there were no significant differences in lithium PK properties between boys and girls. Finally, it should be noted that we did not analyze the potential impact of physiological factors such as the percentage of neutrophils or magnesium and sodium levels on the lithium serum concentration (Xu et al., 2019).

## Conclusion

In summary, we have developed a population PK model of lithium in children with intellectual disability. The proposed model can be used for further PK/PD analysis to optimize the dosage regimen of lithium in children.

## Data Availability

The original contributions presented in the study are included in the article, further inquiries can be directed to the corresponding authors.
